# Examination of the perceived agility and balance during a reactive agility task

**DOI:** 10.1371/journal.pone.0198875

**Published:** 2018-06-13

**Authors:** Leia Stirling, Chika Eke, Stephen M. Cain

**Affiliations:** 1 Department of Aeronautics and Astronautics, Massachusetts Institute of Technology, Cambridge, MA, United States of America; 2 Institute for Medical Engineering & Science, Massachusetts Institute of Technology, Cambridge, MA, United States of America; 3 Department of Mechanical Engineering, Massachusetts Institute of Technology, Cambridge, MA, United States of America; 4 Department of Mechanical Engineering, University of Michigan, Ann Arbor, MI, United States of America; University of Illinois at Urbana-Champaign, UNITED STATES

## Abstract

In vehicle dynamics, it is commonly understood that there is an inverse relationship between stability and maneuverability. However, animal studies have found that stability and maneuverability can coincide. In this study, we examine humans running a reactive agility obstacle and consider the relationship between observational perceived agility and balance, as well as the relationship between quantified surrogates of agility and balance. Recreational athletes (n = 18) completed the agility task while wearing inertial measurement units (IMUs) on their body. The task was also video-recorded. An observational study was completed by a separate group of adults (n = 33) that were asked to view the videos and score each athlete on a Likert scale for balance and for agility. The data from the body-worn IMUs were used to estimate quantified surrogate measures for agility and balance, and to assess if the relationship between the quantified agility and balance was in the same direction as the perceived relationship from the Likert scale responses. Results indicate that athletes that were given a higher Likert agility score were also given a higher balance score (*r*_*s*_ = 0.75,*p* < 0.001). Quantitative surrogates of agility and balance also showed this same relationship. Additional insights on technique for this reactive agility task were informed by the quantitative surrogates. We observed the importance of stepping technique in achieving the faster completion times. The fast performing athletes spent a greater proportion of the task in double support and lower overall time in single support indicating increased periods of static stability. The fast performing athletes did not have a higher body speed, but performed the task with a more efficient technique, using foot placement to enable heading changes, and thus may have had a more efficient path. Similar to animal studies, people use technique to enable agile strategies while also enabling increased balance across the task.

## Introduction

In vehicle dynamics, including aircraft, submarines, and cars, it is commonly understood that there is an inverse relationship between stability and maneuverability. Here maneuverability refers to the ability to turn (i.e., change heading) and specifically considers a series of changes in direction and position for a specified purpose [1]. A vehicle with greater stability will require a larger turn radius and a longer time to complete the turn. While the turn radius is related to size and weight, it is also a function of the underlying vehicle center of gravity location [2]. Human-engineered vehicles that are highly maneuverable are stabilized using automated control systems. Applying this framework to human motion, one might infer that to be agile (having high maneuverability) the human would be less balanced (having lower stability). However, it has been observed that animals in the air, water, and on ground have high agility while maintaining a balanced state [1,3–5]. Animal morphology and motor mechanisms are important factors in the relationship between stability and maneuverability [6]. Before considering these examples in more detail, it is important to discuss stability.

Stability has been formalized by different communities for a variety of applications. Here we consider the definitions of static and dynamic stability in the context of animal or human motion. Static stability (static balance) is when the animal/human’s center of gravity (CG) falls within the base of support [4,7]. Common measures of human postural static stability include having the person either stand on a force plate, or placing motion capture markers on the torso to permit estimating the center of pressure (COP), or sway pattern, during quiet stance. This stabilogram is then used to estimate static stability measures, such as mediolateral sway, anteroposterior sway, COP velocities, area covered, and path length [8,9]. During bipedal locomotion, the CG moves beyond the base of support, thus creating periods of unsteadiness when considering static stability criteria. Dynamic stability requires the modulation of motor patterns to provide proper foot placement such that a fall does not occur [10]. Within the field of dynamical systems, dynamic stability is considered as the ability of characteristic measures of locomotion to return to steady state values after a perturbation [4]. This definition aligns with vehicle dynamic stability, in which dynamic stability is present if the dynamic motions of the vehicle will eventually return the vehicle to its original state [11]. In locomotion, the CG being within the base of support is not a necessary nor sufficient condition for dynamic stability (i.e., instability can occur if the CG is within the base of support and dynamic stability can occur if the CG leaves the base of support). Pai and Patton [12] found that horizontal CG velocity is critical for understanding dynamic stability, with Hof [13] defining an extrapolated CG that incorporates the CG position and velocity to provide insight on regions of dynamic stability. Additional surrogates for dynamic balance during human locomotion include stride length, stride width, stride velocity, percent of time in double support, and the variability in these parameters. The fear of falling in older adults is associated with shorter stride lengths, slower velocity, and increased percent time in double support [14]. Falling is associated with increased variability in these parameters [14,15]. Comparisons between older and younger subjects also highlight that older subjects have shorter step lengths, increased double-support period, and decreased push-off power, which are markers for increased dynamic balance [7], and imply that older subjects may be selecting a motor pattern that provides a reduced chance of falls.

The dynamical systems definition of dynamic stability may be too stringent as Hasan [16] posits that neural responses do not always immediately remove effects of perturbation. Instead they may actually exacerbate the effects of the perturbation, although runaway instability leading to falls can be prevented. Thus, another possible indicator of stability could be a proactive adjustment where voluntary strategy, such as modulating joint stiffness or gait parameters, enables dynamic balance. The change in strategy by the older adults provides a voluntary strategy that enables dynamic balance by increasing steadiness through the use of double support during the task. Acasio et al. [17] found a relationship between lateral margin of stability and peak lateral impulse during a side-stepping task, supporting that planned maneuvers also have a trade-off between passive stability and the force input required to maneuver.

Studies in animals have shown that underlying compliance in the limb structure is important for maintaining balance, and has been modeled through spring-mass systems, e.g. [3,18,19]. The adjustment of limb posture between flexed and extended joints dynamically changes the mechanical advantage of the system. Hedrick et al. [5] also find that flapping animals with higher stability develop the capability to perform particular wing maneuvers that enable maneuverability. These morphological factors make humans and animals different from the rigid vehicle structures.

While the conceptual definition of agility is the ability to quickly change speed or direction, there is not agreement on how agility should be measured and it has been quantified differently for a variety of applications [20]. In the presence of spatial and/or temporal uncertainty Young et al. [21] and Sheppard and Young [20] outline a definition for agility that comprises of perceptual and decision-making factors along with considerations for changing direction with speed, including technique, sprinting speed, muscle qualities, and anthropometry. Reactive agility requires a response to a stimulus during the task, whereas in some agility tasks the waypoints are known *a priori*. Varied activities will naturally require a different set of relevant techniques to achieve the change in direction. Eke and Stirling [22] examined how the definition of agility changed after viewing a specific reactive agility task and observed that additional techniques were articulated by the observers beyond speed, including the path taken and selected body alignment.

In the present study, we examine humans running a reactive agility obstacle and consider the relationship between observational perceived agility and balance, as well as the relationship between quantified surrogates of agility and balance. By perceived balance and agility, we consider the rating an external observer gives an athlete performing the task. We hypothesize there will be a relationship between perceived agility and balance. We then estimate quantified surrogate measures for agility and balance to assess if the relationship between the quantified agility and balance is in the same direction as the perceived relationship. With these data, we assess whether the athletes performing this task simultaneously maintain balance and agility as has been seen in animal studies.

## Materials and methods

### Reactive agility study participants and experimental protocol

The reactive agility study included 18 recreational athletes (9 female, 9 male; age: 20.5±1.9 years; height: 1.7±0.1 m (67.9 ± 4.3 inches); weight: 67.5±13.3 kg (148.8 ± 29.3 pounds); mean ± standard deviation). A subset of athletes (n = 16) had videos that could be used for the observational study. Participants were recruited during September and October 2015 using emails to relevant mailing lists, including those affiliated with the University of Michigan ROTC network and University of Michigan club sports. No participants dropped out of this study. Athlete participants wore 13 wireless IMUs (APDM Opal IMUs, with a 3-axis accelerometer (±6g range), angular rate gyro (±2000 deg/s range), and magnetometer (±6 Gauss)). The IMUs (sampled at 128 Hz) were placed on the feet, shanks, thighs, sacrum, torso, forearms, biceps, and head. Each IMU was secured with elastic straps and athletic tape. For the present analysis of the reactive agility obstacle, data from three IMUs strapped to the sacrum and feet were used. Additional sensors were placed on the participants to assess hypotheses regarding obstacles and metrics not presented here.

**Fig 1:**
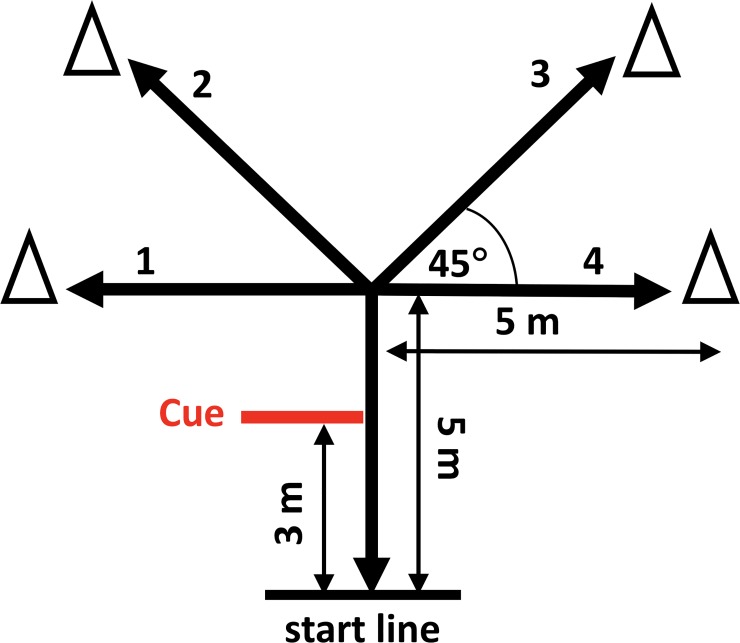
Reactive agility course [23], adapted from Sekulic [24]. Athletes received verbal cues at the location notated and touched 4 endpoint cones per trial. The order of endpoints called out were 4-3-4-1, 2-4-3-4, and 3-4-1-2. Note that not all cones were touched in each order so that there was not an expectation of which cone would be called.

The reactive agility obstacle (Fig 1) was a sub-set of the obstacles performed by the athletes. To complete the obstacle, athletes ran from the start line to an endpoint, touched the top of the endpoint cone, ran back to the start line, and turned around to repeat these actions for three more endpoints as quickly as possible. Endpoints were vocally announced each time the athletes crossed the cue line. Athletes were not provided a strategy on how to complete the task, but were instructed to complete the task as quickly as possible without falling down due to slipping. All athletes provided written informed consent and procedures were approved by the University of Michigan IRB and the MIT Committee on the Use of Humans as Experimental Subjects (COUHES). The videos were parsed and de-identified by blurring athlete faces. The reactive agility videos of the athletes for two times through the obstacle were used within the observational study. Videos were shown at real-speed and not normalized for time.

### Observational study participants and experimental protocol

The observational study was completed by 33 adults (mean age 30 years, SD = 9 years; 16 female, 17 male). Participants were recruited within an expert group—athletic (n = 8, 5 female/3 male), clinical (n = 7, 5 female/2 male), military (n = 8, 1 female/7 male)—or novice group (n = 10, 5 female/5 male) based on their experience evaluating human performance. Recruitment occurred from June–October 2016 and included sending emails to relevant mailing lists, including those affiliated with the MIT ROTC network, clinical collaborators, and MIT athletics. Expert groups were familiar with formal training and evaluation guidelines within their field. The novice group had no previous knowledge of formal guidelines. The athletic group consisted of coaches specializing in football, rugby, soccer, field hockey, tennis, and track. The clinical group consisted of physical therapists. The military group included experienced members of Air Force and Army Reserve Officers' Training Corps (ROTC). Procedures for the observational study were approved by the MIT COUHES and all participants provided written informed consent.

Participants completed an online agility evaluation survey consisting of 4 parts (See S1 Appendix). Part 1 was a short answer question asking for any terms or definitions that the participant associated with agility and balance performance. Part 2 presented the videos showing the 16 athletes completing their second time through the reactive agility course (Section 2.2). Participants were asked to score each athlete’s video on a 7-point Likert scale for balance and for agility, where a score of 1 represented not agile/poor balance and 7 represented highly agile/excellent balance. Each video was approximately 45 seconds long and was presented on a new page of the form in a randomized order. Participants took a 10-minute break after the first 16 videos. The second set of 16 videos showed the athletes completing their third time through the reactive agility course and were presented in mirrored order, without informing participants of the repetition of athletes. There was an option to take a 5-minute break before beginning Part 3 of the survey, which requested a forced ranking of agility performance, which will not be discussed in the present paper. Part 4 of the survey provided space for further explanation if the participant’s definition of agility or balance had changed based on watching the videos. Survey completion time ranged from 1 to 2 hours.

### Data analysis

Previously Eke et al. [23] showed athletes with faster completion times had the smallest number of foot contacts after normalizing by height, highest stride length variance, highest forearm angular velocity variance, and highest stride frequency after normalizing by height. Here we extend considerations of agility by examining the velocity of the sacrum across the task and an estimation of acceleration for each endpoint region. Sacrum velocity provides a quantitative surrogate that enables decoupling body speed and path efficiency from the completion time. An athlete may run at a fast speed, but follow an inefficient path, thereby having a longer completion time. Both speed and path efficiency were terms experts used to describe this reactive agility task [22]. Velocity of the sacrum was estimated by integrating the accelerations and removing drift error using a zero velocity update as described in Ojeda and Borenstien [25]. Velocity was assumed to be zero at the start and stop of the trial, which was consistent with the experimental protocol. Body speed was then estimated as the L2-norm of the three velocity components. From the body speed estimation, we calculate a cone acceleration parameter, defined as

$${\hat{a}}_{cone}=\frac{\left(v_{Post\;Cone\;Peak}-v_{Pre\;Cone\;Peak}\right)}{\left(t_{Pre\;Cone\;Peak}-t_{Pre\;Cone\;Peak}\right)}$$ (1)

where the body speed local maximum prior to and post cone are used to define the turning time. The peaks were determined after a 4^th^ order low-pass filter (1 Hz) of the estimated body speed.

As a surrogate for balance, we examined the time spent in single, double, and no support, as well as the percent time in these phases. These measures were selected as they provide insight on stepping strategy, which Hasan [16] highlights is important for maintaining dynamic balance. Foot contacts (heel-strike and toe-off) were detected using a wavelet analysis to determine when measured foot acceleration and angular velocity contained a threshold level of high frequency [26]. These contact times were used to determine when the left and right foot were in single, double, or no support. To explore where double support occurs, time was normalized between cues and discretized into epochs. Thus the normalized time epochs consisted of the periods from cue 1 to cue 2, cue 2 to cue 3, and cue 3 to cue 4. For this analysis, the time periods from start to cue 1 and from cue 4 to the end were not included. The percent time in double support for an epoch was then the count of the time points in double support across all trials for the selected normalized time epoch divided by the total count of time points within the epoch for all trials. While additional measures of dynamic balance for locomotion include the margin of stability (center of mass location to base of support location) or the extrapolated center of mass [13], these measures were not estimated with the wearable sensors as estimations of position have not been validated for this task with the present IMU accelerometer range.

Correlations between Likert scores for balance and agility, as well as with Completion Time were compared using a Spearman correlation. The relationship between the quantified surrogates of balance and agility were examined using linear regression models and Spearman Correlations (when parametric assumptions were not met). As there was lower interrater reliability for the observational scores, a direct comparison between the quantitative measures and the perceived scores is not presented. Correlations are presented with the correlation coefficient (*r*_*s*_) and p-value. One factor regressions are presented as the slope (*b*), the coefficient of multiple determination (*R*^2^), and p-value. Multifactor regressions are presented with the parameter estimate, t statistic, and p-value.

## Results

### Human perception of balance and agility

**Fig 2:**
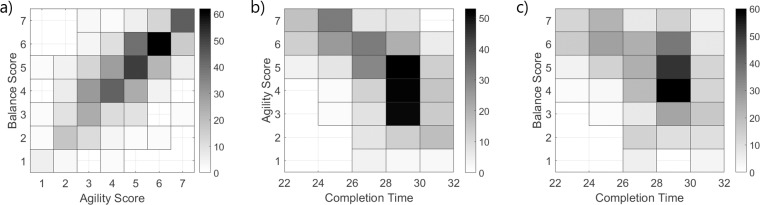
**Two-dimensional histogram for the (a) count of rater agility vs. balance scores, (b) completion time vs. agility score, and (c) completion time vs. balance score**.

Athletes that were given a higher Likert agility score were also given a higher balance score (*r*_*s*_ = 0.75,*p* < 0.001, Fig 2A). Observational raters were not just using completion time to make their assessments, as inferred by the low correlations between completion time and the Likert scores (*r*_*s*_ = −0.57,*p* < 0.001 for Agility Score and Completion Time; *r*_*s*_ = −0.39,*p* < 0.001 for Balance Score and Completion Time). Fast athletes received higher agility and balance scores, while slower athletes received scores along the full spectrum (Fig 2B and 2C). This implies that other technical factors were considered in the selection of the perceived score.

### Quantification of agility

**Fig 3:**
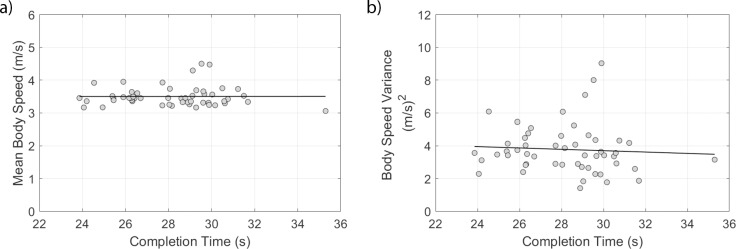
(a) Mean body speed and (b) variance in body speed with respect to time. The relationships were not significant between mean body speed or variance in body speed with completion time.

**Fig 4:**
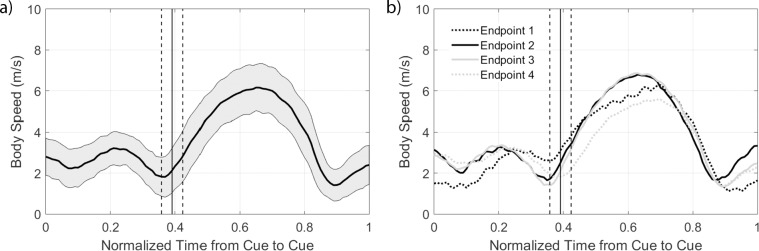
Body speed estimated using the sacrum IMU vs. normalized time. The time was normalized from one cue to the next cue. The solid vertical line indicates the mean location of the cone within the normalized time period, with the dashed lines showing one standard deviation from the mean. (a) Mean body speed across all endpoints. The shaded gray region indicates ±1 standard deviation from the mean body speed, where the mean was calculated across 156 cue-cue time periods (52 trials, each with 3 cue-to-cue regions). (b) Mean body speed segmented by endpoint, standard deviations not shown for ease in disambiguating the mean lines.

**Fig 5:**
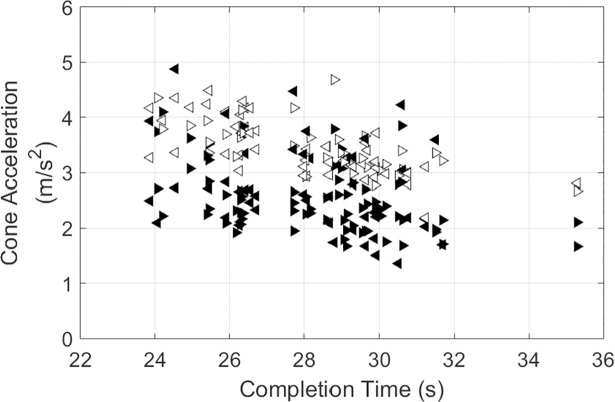
Estimated cone region acceleration. The body speed local maximum prior to and post cue were used to define the turning time. The acceleration is the ratio between the difference in velocity at these peaks divided by the difference in time between these peaks. Triangles that are white-filled are the inner cones (Endpoints 2 and 3) and black-filled are the outer cones (Endpoints 1 and 4). Triangles pointing to the left are turns to the left (Endpoints 1 and 2) and pointing to the right are right turns (Endpoints 3 and 4).

There was no significant correlation between mean body speed and completion time (*r*_*s*_ = 0.04,*p* = 0.764) or body speed variance and completion time (*r*_*s*_ = −0.12,*p* = 0.381) (Fig 3). The changes in body speed were aligned with locations in the course, with slower speeds occurring prior to the cue and at the cone (Fig 4). The largest speeds were observed during the period where the athlete ran from the cone to the start line and then slowed down as they approached the next cue. The peak velocity post-cone was smaller for the outer cones (Endpoints 1 and 4) than the inner cones (Endpoints 2 and 3). These results impact the estimated cone acceleration (Fig 5, Table 1), which highlights that there was a greater estimated acceleration into and out of the cone region for participants with shorter completion times. A greater estimated acceleration was observed for the inner cones (Endpoints 2 and 3) with respect to the outer cones (Endpoints 1 and 4). There was no effect of whether the turn was to the left or right or for the interactions between these independent variables. For this multiple linear regression, the underlying assumption of constant variance was met, but the data were skew (normality was not met). Here the untransformed data are presented in Fig 4, with the multiple regression assessed using a log transformation to remove the skew (Table 1).

**Table 1:** Estimated regression coefficients for the log of the cone acceleration dependent variable.

Parameter	Estimate	t Statistic	p-value
**Intercept**	2.214	13.64	<0.0001
**Completion Time**	-0.040	-7.01	<0.0001
**Left-Right Endpoints**	-0.017	-1.16	0.249
**Inside-Outside Endpoints**	-0.146	-10.81	<0.0001

### Quantification of balance

**Fig 6:**
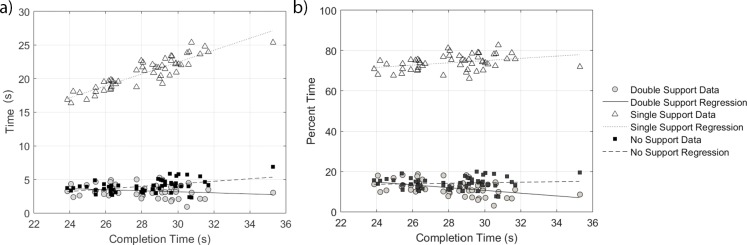
(a) Absolute time in double, single, and no support phases. (b) Percent of time spent in double, single, and no support phases.

**Fig 7:**
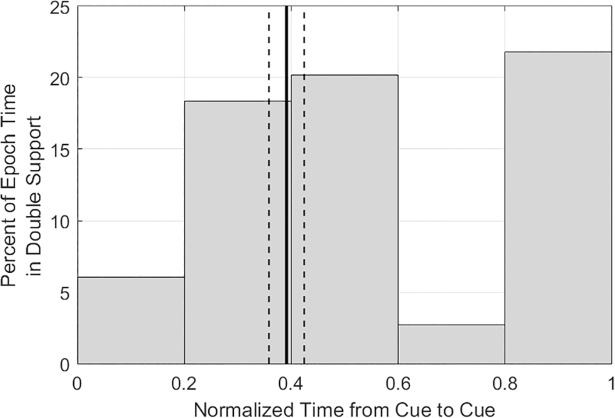
Bars indicate the cumulative percent time of double support between cues 1/2, cues 2/3, and cues 3/4 for all 18 athletes over the repetitions of the course (total of 52 runs through the course). The time was normalized from one cue to the next cue. The vertical line indicates the mean location of the cone within the normalized time period, with the dashed lines showing one standard deviation from the mean.

With increasing completion time (Fig 6A), there was a significant increase in single support time (*b* = 0.887, *R*^2^ = 0.810, *p* < 0.0001), no significant difference in double support time (*R*^2^ = 0.037, *p* = 0.173), and a significant increase in no support time (*b* = 0.189, *R*^2^ = 0.217, *p* < 0.001). When considering the duration of gait phases as percentages of trial time (Fig 6B), these data indicate increasing completion time occurs with a significant decrease in percent time in double support (*b* = −0.687, *R*^2^ = 0.192, *p* = 0.001), a significant increase in percent time in single support (*b* = 0.562, *R*^2^ = 0.123, *p* = 0.011), and no significant difference in percent time in no support (*R*^2^ = 0.010, *p* = 0.475). The double support regions tended to occur around the cone and prior to the cue (Fig 7). Lower double support was observed just after the cue and in the epoch between the cone and cue.

### Relationship between quantified agility and balance

**Fig 8:**
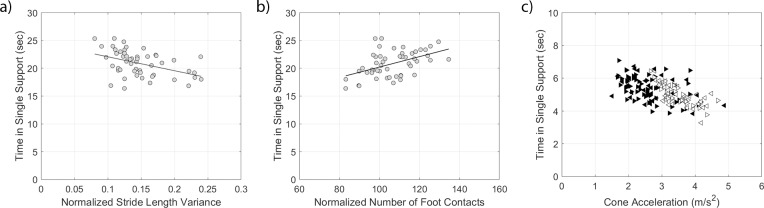
**Relationship between time in single support and (a) the normalized stride length variance, (b) the normalized number of foot contacts, and (c) cone acceleration.** One outlier trial was removed from the normalized stride length variance plot and correlation.

A longer single support time was indicative of decreased balance. Increased normalized stride length variance and decreased normalized number of foot contacts were previously shown to be indicative of higher agility task completion time [23]. In the present analysis, it was observed that increased cone acceleration was related to decreased completion time. With increased normalized stride length variance (Fig 8A), there was a significant decrease in the single support time (*r*_*s*_ = −0.44, *p* = 0.002). With an increased normalized number of foot contacts (Fig 8B), there was a significant increase in the single support time (*r*_*s*_ = 0.46, *p* < 0.001). With increased cone acceleration, there was a decrease in the single support time (*R*^2^ = 0.33,*F* = 24.47,*p* < 0.0001, Fig 8C, Table 2). No significant effect was observed for endpoint location for this relationship. Thus all three comparisons support that a higher agility quantitative surrogate is associated with a higher balance quantitative surrogate.

**Table 2:** Estimated regression coefficients for the single support time with cone acceleration and endpoint location.

Parameter	Estimate	t Statistic	p-value
**Intercept**	7.039	28.06	<0.0001
**Cone Acceleration**	-0.606	-7.59	<0.0001
**Left-Right Endpoints**	-0.007	-0.13	0.900
**Inside-Outside Endpoints**	-0.085	-1.44	0.153

## Discussion

Observational perceived agility and balance, as well as quantified surrogate measures of agility and balance were investigated. We hypothesized there would be a relationship between perceived agility and balance. We then estimated quantified surrogate measures for agility and balance to assess if the quantified surrogate measures had similar relationships to the perceived scores. While in vehicle dynamics there is an inverse relationship between balance and agility, our data support the findings of animal studies that higher agility is possible while maintaining a balanced state [1,3–5].

The data support a positive correlation between perceived balance and agility. The observers scored those with higher agility as also having higher balance. While common to measure agility through a completion time, the data showed a low correlation between agility score and completion time. Athletes with fast completion times had higher agility scores; however, athletes with slower completion times had scores that spanned the available range. These data indicate that observers use additional cues beyond completion time when considering agility, especially for the lower performers. Similarly, balance had a low correlation with completion time overall, where athletes with faster completion times had higher balance scores, but athletes with slower completion times had a wide range of scores. Here we consider increased operational performance as a lower completion time. As performance increased, high agility and high balance converge, whereas lower performers had increased score variance.

Previously Eke and Stirling [22] found that athletes with faster completion times had a smaller fourth spread (also called the interquartile range) in the observational scores provided. Thus, we consider the techniques that enabled faster completion times as a surrogate for agility. However, we recognize that techniques to achieve these completion times may vary and that alternate strategies may exist if task parameters are changed. Eke et al. [23] showed faster completion times with techniques that included having a smaller number of foot contacts after normalizing by height, higher stride length variance, higher forearm angular velocity variance, and higher stride frequency after normalizing by height. Additional surrogates for agility examined here included the mean body speed of the athlete and the acceleration in the endpoint cone region. Interestingly, mean body speed was not correlated with the completion time. Athletes with slower completion times were not moving at a slower average speed. Estimating distance traveled as completion time multiplied by mean body speed indicates that athletes with longer completion times traveled a greater distance. The less efficient paths could be due to the increased single support usage creating a larger radius turn. In the current data set, we cannot decouple whether the less efficient path was due to the stimulus response (cognitive) or motor action (physical) component. A follow-up study examining the same course in a planned versus reactive implementation would enable this disambiguation. The increase in acceleration in the endpoint cone region with faster completion times is consistent. A greater acceleration was observed for the inner endpoints, highlighting that when the turn angle was smaller, participants had a greater change in velocity over the time period. Chang and Kram [27] have shown that maximum running speed during curves is limited by the ground reaction forces necessary to attain maximum velocities compared to straight running. Thus, the higher body speeds attained in the present study during turns with lower curvature is consistent with the literature.

Athletes were also observed to have a non-constant velocity during the task, which is consistent with the hexapod study of Jindrich and Full [3] where COM had non-constant velocity during maneuverability tasks. The hexapods were observed to have lower velocities during regions of high curvature. Here we found athletes had lower speeds from the cue to the cone region, with higher velocities from the cone to the next cue. While literature state that turns require straight-ahead running punctuated by periods where the heading was deflected [3,4], we did not estimate the trajectories of the body as an additional integration was needed to estimate position. Performing this integration would create additional positional drift and correcting this drift is still an open research question. The heading of the sacrum was previously calculated [23], and for this task, we did not observe many periods of constant heading between the cue and the cone. While misalignment between the angle of deflection and the angle of body rotation is important for resuming straight running following a single turn [3], the measure is confounded in this study as athletes purposely over or under-rotated at the cones based on their foot placement as they prepared for the next planned rotation in the task.

To investigate dynamic balance, we examined the surrogates of phase of gait timing (single, double, no support). A very high positive correlation was observed between time in single support and completion time, with a low positive correlation between time in no support and completion time. These differences in time create an increase in percent time spent in single support and a decrease in percent time spent in double support for athletes with longer completion times. Thus, we observe that those with slower completion times spend more time in a statically unstable single support phase. These data support the observational perception of improved balance for the athletes with faster completion times.

When pooling all athletes together, we observe that double support tends to occur prior to the cue and near the cone. The increased usage of double support prior to the cone may enable the athlete to more easily shift which foot could take on the load from the body when the cue was received, thus enabling a more efficient path and requiring fewer overall steps to change direction. Bauby and Kuo [28] find that active control required for lateral stability during human gait is commonly obtained through step width corrections, while more passive mechanisms can be used in steady straight walking (step length). While we do not estimate step width in the present study, the increased percentage of the task spent in double support provides periods with greater lateral stability during the task.

The quantified surrogate measures for balance and agility support the perceived relationships from the subjective scores. Normalized stride length variance decreased, normalized number of foot contacts increased, and cone acceleration decreased with increases in single support time. Although the relationship between the perceived balance and agility (*r*_*s*_ = 0.75) was greater than the relationship for the quantified surrogates (*r*_*s*_ = −0.44 for normalized stride length variance, *r*_*s*_ = 0.46 for normalized number of foot contacts, and *r* = 0.57 for cone acceleration).

In this study, we observe the importance of stepping technique in achieving the faster completion times. The importance on technique is similar to what was observed in flapping flight by Hedrick [5]. People change strategy to obtain the necessary maneuverability characteristics. Young and James [21] provide evidence that muscle strength (as measured through concentric leg extension power) was not significantly related to performance in change-of-direction sprints. It may be that while there is an underlying strength needed to perform quick turns, that technique has a larger influence on overall performance.

The selected surrogates for balance were based on the gait phase timings. A limitation to the selection of these surrogates is that these parameters are related to the selected gait speed [29]. As the mean body speed was not significantly different across the completion times, this selection should not affect the interpretation of the results. The balance surrogates selected enabled an understanding of static stability and dynamic balance. With improvements in IMU position estimation, additional surrogates of dynamic stability could be further examined. An additional limitation to the cone acceleration metric may have been due to the 128 Hz sampling rate, which would limit the precision of the time estimation. However, this sampling rate was sufficient for the reactive agility task performed here.

In this task, there was a lower risk of falls due to the environment. While the course was performed outdoors, yielding variations in ground friction, the ground was mostly level. Had the task been performed over rocks or on a balance beam, the level of risk would be increased and the techniques may have changed. For example, in a balance beam task requiring turns, more precision is required in foot placement and there is a higher risk of falls when over or undershooting the desired foot placements. Fitts and Peterson [30] showed for reaching motions that the movement time increases with increased precision placement requirements. For this agility task, dynamic balance could be maintained with additional steps if necessary. However, an agility task that requires greater underlying dynamic balance precision may generate a different relationship between agility and balance. Cain et al. [26] found that in a balance beam task increases in load (which would indicate increased risk if there was a fall) were observed with an increase in double support time. Follow-up work should investigate surrogates of agility within the balance beam task as well.

This task also included a cognitive element as it was a reactive agility course. The technique implemented during a planned version of the agility course, where the endpoints are provided at the start of the trial and not at each passing of the cue line, would naturally create a different foot placement. The need to responsively shift center of mass over a specific foot to change direction is not required in a planned task as direction is known *a priori*. Thus the cognitive element of on-line vs. *a priori* motion planning may also affect the relationship between agility and balance.

## Conclusion

In vehicle dynamics, it is commonly understood that there is an inverse relationship between stability and maneuverability. However, studies of animals in the air, water, and on ground have observed high agility while maintaining a balanced state. In this study of a reactive agility task, a positive relationship was found between perceived agility and balance. The estimated surrogate measures for agility and balance were then examined to determine if perception was aligned with actual strategy techniques. It was observed that fast task completions times were associated with higher agility and balance scores. The fast performing athletes spent a greater proportion of the task in double support and lower overall time in single support indicating increased periods of static stability. The fast performing athletes did not have a higher body speed, but performed the task with a more efficient technique, using the foot placement to enable heading changes, and thus may have had a more efficient path. Similar to animal studies, people can use technique to enable an agile strategy while also enabling increased dynamic balance across the task. These conclusions must be interpreted in the context of the selected reactive agility task.

## Supporting information

S1 Appendix Survey information. This file contains additional details on the questions asked during the observational study.
(PDF)

S1 Dataset Data tables. This file contains the quantified data for each subject for each metric from the reactive agility study and the perceived agility and balance scores for each rater from the observational study.
(XLSX)
