# Supplementary material for: Examination of the perceived agility and balance during a reactive agility task
Source: PLoS One. 2018 Jun 13;13(6):e0198875. doi: 10.1371/journal.pone.0198875 (PMC5999270; doi:10.1371/journal.pone.0198875)
Supplement: S1 Appendix — This file contains additional details on the questions asked during the observational study. (PDF) [file pone.0198875.s001.pdf]

S1 Appendix. Survey Information

Participants completed a 4-part online agility and balance evaluation survey. Part 1 of the survey was a short answer section including the following two questions:

1. Which terms or definitions do you associate with agility?
2. Which terms or definitions do you associate with balance?

Participant responses were entered in a text box with no character limit. Upon completing both questions, participants proceeded to Part 2 of the survey and viewed the following instruction:

"Please use your definitions of agility and balance to score the individual below on a scale ranging from 1 to 7. Provide reasoning for the score in the space below the video."

Participants viewed and scored a total of 32 videos, with a 10-minute break given after the first 16 videos. A text box with no character limit was provided under each video to allow score explanations. Here is an example video screen shot with associated scoring scales:

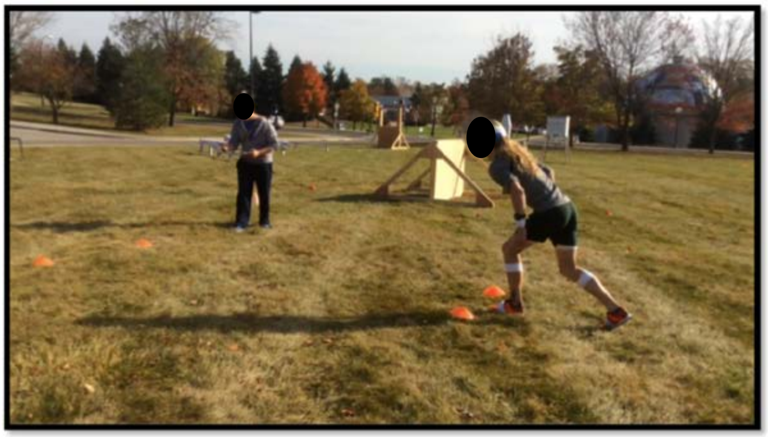

	1	2	3	4	5	6	7	
Not Agile	○	○	○	○	○	○	○	Highly Agile

	1	2	3	4	5	6	7	
Poor Balance	○	○	○	○	○	○	○	Excellent Balance

After completing Part 2, participants were given the option to take a 5-minute break before proceeding to Part 3. The third part of the survey began with the following instruction:

"Please use your definitions of agility and balance to rank the videos. For each set, the video with the most agile/balanced individual should receive a rank of 1. The video with the least agile/balanced individual should receive a rank of 5 (no ties permitted). Provide reasoning for your ranking in the space below the videos."

Participants viewed 2 subsets of 5 videos. Each subset was shown on a new survey page with a text box (no character limit) for rank explanations. An example subset is shown below:

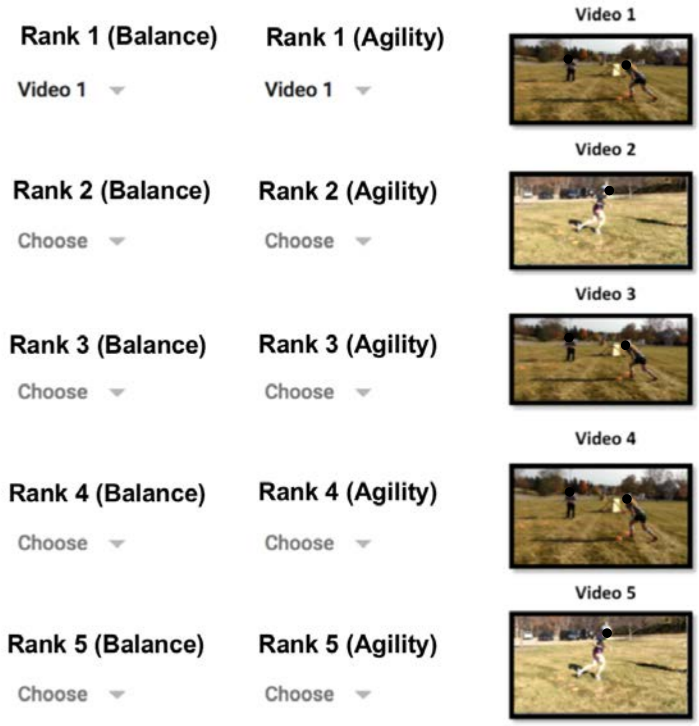

Part 4 of the survey consisted of a short answer section that allowed participants to provide further explanation for their definitions of agility and balance by answering the following 2 questions. No character limits were enforced.

1. How has your definition of agility and/or balance changed after watching the videos?
2. Are there any factors you view as important for agility and/or balance that the course shown in the videos did not test? If there are, please list these factors and any suggestions you may have for improvement.
